# Facial rash with exuberant crusting in pregnancy

**DOI:** 10.1097/JW9.0000000000000055

**Published:** 2022-09-26

**Authors:** Raymond Zhao, Severine Cao, Frank Wang

**Affiliations:** a Department of Dermatology, University of Michigan Medical School, Ann Arbor, Michigan

## Case Summary

**Fig. 1.**
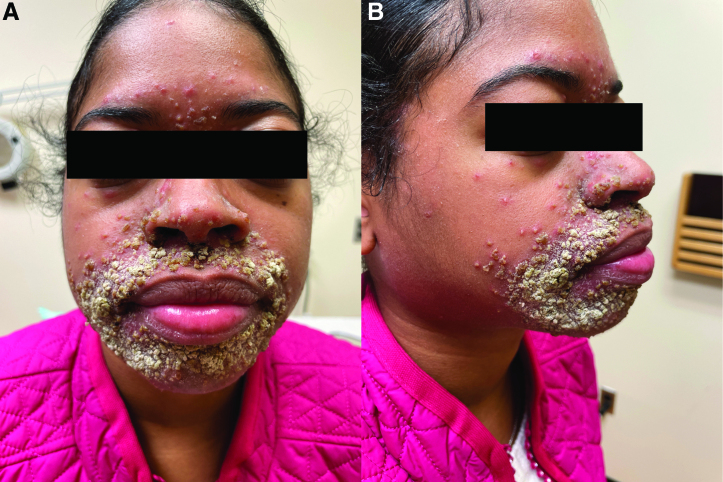
Clinical presentation of the (A) frontal and (B) right face showing erythematous papules and pustules coalescing into plaques with thick, yellow-gray crust around the nose and mouth with sparing of the vermilion border and extension onto the glabella.

A woman in her 20s with a history of systemic lupus erythematosus controlled with azathioprine and low-dose prednisone presented at ten weeks of pregnancy after developing pruritic, painful facial lesions over three weeks. She had no history of rosacea, topical corticosteroid use, or recent fevers, but was wearing masks frequently. On examination, she appeared non-toxic and had numerous coalescing erythematous papules and pustules with yellow-gray crusting around her nose, mouth, and glabella (Fig. 1). The vermilion border was spared. No comedones were present. Herpes simplex virus polymerase chain reaction (PCR), varicella zoster virus PCR, bacterial cultures, and fungal cultures were negative. Punch biopsy of a pustule showed intraepidermal neutrophilic spongiosis with subcorneal pustules, diffuse dermal neutrophilic inflammation, and dermal edema.

## Question 1

### Which of the following is the most likely diagnosis?

A. Pyoderma faciale
B. Impetigo
C. Acute lupus flare
D. Pyostomatitis vegetans
E. Amicrobial pustulosis associated with autoimmune disease (APAD)

**Correct answer:** A. Pyoderma faciale. Disease is characterized by numerous erythematous nodules, papules, and pustules erupting on the central face.

She was diagnosed with periorificial pyoderma faciale. With her obstetrician’s approval, we initiated a three-month course of cephalexin, in line with standard treatment durations for rosacea. At two months follow-up, there was near-resolution.

## Discussion

Pyoderma faciale, also known as rosacea fulminans, is a rare inflammatory facial dermatosis most commonly affecting women between 20 and 40 years old with a history of flushing or rosacea.^1^ Approximately 10% of cases occur during pregnancy, and around 40% can be triggered by menopause, medication changes, or emotional stressors.^1^ Patients present suddenly with tender coalescing inflamed pustules, nodules, draining sinuses, and cysts/abscesses on the central face.^2^ Our patient presented with numerous pustules, with the additional and unique feature of thick, confluent, yellow-gray crusting. To our knowledge, prior reports of patients with crusted papulopustules have not shown such exuberant facial crusting.^3^ The reasons for our patient’s dramatic presentation are unclear. Possibly, her underlying systemic lupus erythematosus (SLE), stress and altered immunity during pregnancy, and mask wearing promoted vascular hyperreactivity and inflammation predisposing to development of pyoderma faciale.^2^

Pyoderma faciale can mimic infectious and inflammatory processes. In our patient with SLE on immunosuppression, the differential diagnosis included infections such as impetigo, an acute lupus flare, pyostomatitis vegetans, or an atypical facial presentation of amicrobial pustulosis associated with autoimmune disease (APAD). Given the patient’s pregnancy status, morphology of lesions with centrofacial distribution and sparing of the vermilion border, non-toxic appearance, stable SLE and negative infectious work-up, pyoderma faciale was felt to be the best diagnosis. Notably, the perioral and perinasal distribution of the rash resembled periorificial dermatitis, which is on the rosacea spectrum, much like pyoderma faciale.

Typically, pyoderma faciale is treated initially with systemic corticosteroids to rapidly reduce inflammation, followed by longer courses of isotretinoin or tetracyclines to maintain improvement. In our patient, the latter 2 treatment options were contraindicated due to pregnancy. Beta-lactam antibiotics can improve pyoderma faciale, although they often fail to achieve resolution as monotherapy and are typically given in conjunction with corticosteroids or isotretinoin.^1^ Recently, erythromycin monotherapy has shown promising results in pregnant individuals.^1^ Our case provides evidence that a robust presentation of pyoderma faciale with a periorificial distribution can be treated safely and successfully with cephalexin monotherapy during pregnancy.

## Question 2

### What dermatologic disorder is most associated with pyoderma faciale?

A. Atopic dermatitis
B. Acne
C. Rosacea/flushing
D. Impetigo
E. Folliculitis

**Correct answer:** C. Rosacea/flushing. Walsh et al.^1^ demonstrated that history of rosacea/flushing is present in 80% of cases of pyoderma faciale.

## Conflicts of interest

None.

## Funding

None.

## Author contributions

Raymond Zhao contributed to the drafting of the manuscript, figure design, and background research. Severine Cao and Frank Wang contributed to the conception and overall planning of the project, supervision of the project, and critical revision of the manuscript.

## Study approval

N/A.

## References

[1] Walsh RK Endicott AA Shinkai K. Diagnosis and treatment of Rosacea fulminans: a comprehensive review. Am J Clin Dermatol 2018;19:79–86. 28656562 10.1007/s40257-017-0310-0

[2] Ribeiro LB Ramos-e-Silva M. Rosacea fulminans. Cutis 2013;92:29–32. 23961522

[3] Debroy Kidambi A Tiffin NJ Ramsay HM. Atypical rosacea in a male patient: case study. Dermatol Online J. 2016;22(2):13030/qt8x65r1kx. 27267199

